# A Microwave Photonic 2 × 2 IBFD–MIMO Communication System with Narrowband Self-Interference Cancellation

**DOI:** 10.3390/mi15050593

**Published:** 2024-04-29

**Authors:** Ying Ma, Fangjing Shi, Yangyu Fan

**Affiliations:** 1 School of Electronic Information Engineering, Xi’an Technological University, Xi’an 710032, China; maying@xatu.edu.cn; 2 School of Electronics and Information, Northwestern Polytechnical University, Xi’an 710129, China; fan_yangyu@nwpu.edu.cn

**Keywords:** microwave photonics, self-interference cancellation, in-band full duplex, multiple input multiple output

## Abstract

Combined in-band full duplex-multiple input multiple output (IBFD–MIMO) technology can significantly improve spectrum efficiency and data throughput, and has broad application prospects in communications, radar, the Internet of Things (IoT), and other fields. Targeting the self-interference (SI) issue in microwave photonic-based IBFD–MIMO communication systems, a microwave photonic self-interference cancellation (SIC) method applied to the narrowband 2 × 2 IBFD–MIMO communication system was proposed, simulated, and analyzed. An interleaver was used to construct a polarization multiplexing dual optical frequency comb with a frequency shifting effect, generating a dual-channel reference interference signal. The programmable spectrum processor was employed for filtering, attenuation, and phase-shifting operations, ensuring amplitude and phase matching to eliminate the two self-interference (SI) signals. The simulation results show that the single-frequency SIC depth exceeds 45.8 dB, and the narrowband SIC depth under 30 MHz bandwidth exceeds 32.7 dB. After SIC, the desired signal, employing a 4QAM modulation format, can be demodulated with an error vector magnitude (EVM) as low as 4.7%. Additionally, further channel expansion and system performance optimization are prospected.

## 1. Introduction

With the development of emerging technologies such as cloud computing, big data, high-definition video, and virtual reality, alongside the continuing proliferation of applications such as the Internet of Things (IoT), artificial intelligence, intelligent transportation, telemedicine, and distance education, the volume of communication data has experienced continuous growth. Communication technologies are evolving towards large-capacity, low-latency, and high-reliability solutions. Advanced communication technologies such as multiple input multiple output (MIMO), quantum communication [1], network slicing [2], edge computing, and edge communication [3] have been actively explored by researchers. Among them, MIMO technology utilizes multiple antennas to simultaneously transmit multiple independent data streams under limited spectrum resources, thereby augmenting spectrum efficiency and data throughput while bolstering resilience against multipath interference. MIMO technology finds indispensable applications across contemporary mobile communications, wireless local area networks (WLANs), radar systems, satellite communications, and diverse interdisciplinary domains. Nevertheless, existing MIMO systems confronting the trend towards large-scale arrays present several challenges: (1) the number of hardware links, including antennas and radio frequency (RF), increases, which increases the cost and complexity of system design; (2) the enormous demand for channel estimation and feedback has intensified the pressure on matrix calculation and signal processing; (3) the complex sources of interference pose higher requirements for the system’s interference management capability; and (4) the presence of multipath fading and signal channel attenuation necessitates large-scale array systems to employ more efficacious compensation and signal processing methodologies. Furthermore, MIMO systems entail heightened power requirements to sustain the operation of multiple antennas and link sets, thereby exacerbating system power consumption and introducing electromagnetic interference among the links.

Incorporating in-band full duplex (IBFD) technology into MIMO systems represents a promising approach to mitigate the hardware implementation challenges associated with large-scale MIMO systems while concurrently optimizing system capacity and array dimensions. Primarily, simultaneous transmission and the reception of co-frequency signals on a single physical link can halve the actual hardware expenditure of a MIMO system. Meanwhile, the inherent doubled spectral efficiency of IBFD technology can further optimize the capacity advantage of MIMO systems. Moreover, IBFD technology can reduce the delay of MIMO communication systems, which is helpful for delay-sensitive applications such as real-time communication [4]. The integration of these two technologies enhances the flexibility of the communication system, facilitating superior adaptation to diverse communication environments and network conditions, consequently enhancing the user experience. IBFD technology presents a relatively mature solution for addressing co-frequency self-interference. Although the IBFD–MIMO system may face more severe interference problems, it can be expanded on existing SIC methods to enhance the effectiveness of interference management. Lastly, in response to the demand for system performance improvement, the MIMO system also has a variety of optimization auxiliary algorithms. In the future, integration with SIC algorithms can be considered to achieve multi-dimensional system performance optimization.

This paper primarily addresses the topic of self-interference cancellation (SIC) in IBFD–MIMO systems. According to the location of the self-interference signal, SIC can be divided into three types: antenna domain cancellation, analog domain cancellation, and digital domain cancellation. These encompass antenna domain SIC technologies such as increasing the physical distance between transmitting and receiving antennas, employing cross-polarized antennas, and implementing placing absorbing shields [5,6]; analog domain SIC technologies such as multi-tap filter networks [7] and vector modulation [8]; and digital domain SIC technologies such as channel estimation [9] and adaptive filtering. However, electronic bottlenecks limit the above schemes in terms of operating frequency bands and bandwidth enhancement. Especially when extending these methods to MIMO systems, additional interference problems between channels will be introduced, which poses a challenge to backend digital signal processing.

In recent decades, microwave photonic SIC technology has been widely studied due to the advantages of the high-frequency band, low loss, large bandwidth, and anti-electromagnetic interference. Based on the principle of reference signal reconstruction, researchers initially constructed parallel optical paths with incoherent light sources, then modulated the received signal and reconstructed the signal. Interference cancellation is finally achieved after adjusting the parameters to meet the phase inversion conditions [10,11,12,13]. As device integration advances, the use of coherent light sources and devices such as electro-absorption modulators (EAMs) [14], dual-parallel Mach–Zehnder modulators (DPMZMs) [15], and polarization division multiplexing Mach–Zehnder modulators (PDM-MZMs) [16], in conjunction with the reference signal reconstruction process for achieving SIC, has progressively gained prominence. Especially when facing the multifunctional integrated RF front-end and long-distance fiber transmission application requirements, the integration of SIC with down-conversion [17,18], periodic power fading suppression [19,20], image interference rejection [21,22], and other functions has become a research hotspot. Concurrently, schemes incorporating multipath SIC functions have also been proposed in [23] to address the intricacies and variability of signal propagation paths. The cancellation depth within the 200 MHz bandwidth range can reach 40 dB. Apart from the analog SIC method based on the dual-driver Mach–Zehnder modulator (DDMZM) [24], an adaptive filter employing the least mean squares (LMS) algorithm is constructed as a pre-equalizer to dynamically adapt and generate a signal closely resembling the multipath SI signal.

It is evident that all the above schemes are only for single-channel transmission cases. In [25], SIC was introduced into the MIMO system for the first time, and a microwave photonic SIC scheme under the IBFD communication system incorporating a 2 × 2 MIMO antenna system was proposed. The cancellation of SI signals between two adjacent transmission antennas in the IBFD system was successfully verified through simulation. However, the real advantages of microwave photonic technology remain constrained by the reliance on multiple electrical delay lines and attenuators for multi-channel reference signal reconstruction. Additionally, a multi-channel optical tunable delay line (OTDL) and optical attenuation module with a wide tuning range and high tuning accuracy were meticulously designed [26]. This scheme eliminated SI signals through wavelength division multiplexing (WDM) technology in a 3 × 3 MIMO radio over a fiber system, achieving an SIC depth of 20 dB at an instantaneous bandwidth of 2 GHz.

Like most communication systems, current microwave photonic SIC systems are also seeking breakthroughs in bandwidth. Nevertheless, there is a high demand for device connectivity coupled with relatively low data rate requirements for special applications such as the IoT, Internet of Vehicles, indoor positioning, and navigation. At this time, increasing bandwidth may not be the foremost priority. Consider, for instance, specific IoT applications enabled by 5G New Radio (NR) technology. These applications typically entail small data packets, utilize high-frequency bands, and require the connectivity of numerous devices with low power consumption. Therefore, narrowband IBFD communication based on the MIMO system has emerged as a better choice. However, the interference situation of the IBFD–MIMO system is relatively complex, particularly due to the inherent SI problem of the IBFD system, which is further compounded under the MIMO system configurations. In such application scenarios, it is essential to eliminate the SI signal of this channel and the interference signal between adjacent antennas to ensure the quality of the received signal. The narrowband SIC method based on microwave photonic MIMO systems can effectively alleviate the multi-channel delay and attenuation adjustment pressure of broadband SIC systems, and effectively eliminate interference signals in the context of narrowband MIMO communication applications.

In view of the narrowband MIMO communication background, a microwave photonic SIC method is proposed, and the simulation verification results are presented in this paper. Polarization multiplexing technology was used to achieve independent dual-channel receiving signal transmission, while a dual-channel reference path was constructed based on optical frequency comb (OFC) and single sideband modulation (SSB) technology. The programmable spectral processor was used to achieve independent amplitude and phase matching of each reference branch in the optical domain. Subsequently, the SI signal could be eliminated through the final balanced photodetection (BPD). In addition to solving the SI problem of single-antenna channels, the proposed scheme can eliminate interference signals leaked by adjacent antennas. It also provides ideas for channel expansion and performance optimization for deploying large-scale MIMO antenna arrays. This holds significant implications for integrating microwave photonic SIC technology into forthcoming communication networks, radar systems, aerospace communications, and related domains. Compared with existing research, the proposed scheme uses a programmable spectrum processor instead of an electric delay line and an electric attenuator group. This single device enables simultaneous optical filtering, amplitude matching, and phase matching, thereby streamlining the system architecture and harnessing the inherent benefits of microwave photonic technology.

## 2. Principles

**Figure 1:**
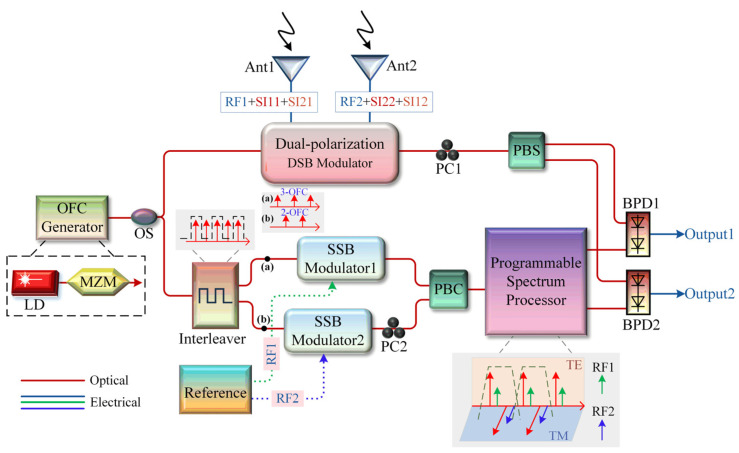
Schematic diagram of the microwave photonic SIC scheme applied to the narrowband 2 × 2 IBFD–MIMO communication system.

Figure 1 presents a schematic diagram of the proposed microwave photonic SIC scheme for the IBFD communication system applied to the narrowband 2 × 2 MIMO antenna system. The system consists of an OFC generator, an optical beam splitter (OS), an interleaver, a dual polarization double sideband (DSB) modulator, two SSB modulators, two polarization controllers (PCs), an optical polarization beam splitter/beam combiner (PBC/PBS), a programmable spectral processor, and two BPDs. In the structure shown in Figure 1, RF1 and RF2 represent the desired received signals of receiving antenna 1 and receiving antenna 2, respectively. SI11 represents the interference signal leaked from transmitting antenna 1 to receiving antenna 1, while SI12 represents the interference signal leaked from transmitting antenna 1 to receiving antenna 2. Similarly, SI22 represents the interference signal leaked from transmitting antenna 2 to receiving antenna 2, and SI21 represents the interference signal leaked from transmitting antenna 2 to receiving antenna 1.

There are various methods for OFC generation [27]. In this scheme, the OFC generator consists of a laser diode (LD) and a Mach–Zehnder modulator (MZM). The LD generates a continuous laser signal $E_{c}\left(t\right)=E_{c}e^{j\omega_{c}t}$ as an optical carrier, and the RF modulation signal $V_{0}\left(t\right)=V_{0}\cos\omega_{0}t$ drives the MZM to generate multiple optical sidebands. $E_{c}$ and $\omega_{c}$ represent the amplitude and angular frequency of the optical carrier. $V_{0}$ and $\omega_{0}$ represent the amplitude and angular frequency of the RF drive signal. The output of the MZM can be expressed as:

(1) $$\begin{array}{cc} E_{MZM}\left(t\right) & =\frac{E_{c}\left(t\right)}{2}\sqrt{\mu_{0}}\left[e^{j\varphi_{0}/2}e^{jm_{0}\cos\omega_{0}t}+e^{-jm_{0}\cos\omega_{0}t}e^{-j\varphi_{0}/2}\right]\\ & \approx \sqrt{\mu_{0}}E_{c}\left(t\right)\left[\begin{array}{l} \cos\left(\varphi_{0}/2\right)J_{0}\left(m_{0}\right)\\ -\sin\left(\varphi_{0}/2\right)J_{1}\left(m_{0}\right)\left(e^{j\omega_{0}t}+e^{-j\omega_{0}t}\right)-\cos\left(\varphi_{0}/2\right)J_{2}\left(m_{0}\right)\left(e^{j2\omega_{0}t}+e^{-j2\omega_{0}t}\right)\\ +\sin\left(\varphi_{0}/2\right)J_{3}\left(m_{0}\right)\left(e^{j3\omega_{0}t}+e^{-j3\omega_{0}t}\right)+\cos\left(\varphi_{0}/2\right)J_{4}\left(m_{0}\right)\left(e^{j4\omega_{0}t}+e^{-j4\omega_{0}t}\right) \end{array}\right] \end{array}$$

where $\mu_{0}$ is the insertion loss of the MZM; $m_{0}$ is the modulation index; and $\varphi_{0}$ is the DC bias angle of the MZM. $J_{n}\left(\cdot \right)$ represents the first kind of *n*-order Bessel function, and under small-signal modulation, the high-order sidebands are ignored. When $\cos\left(\varphi_{0}/2\right)J_{0}\left(m_{0}\right)=\sin\left(\varphi_{0}/2\right)J_{1}\left(m_{0}\right)=\cos\left(\varphi_{0}/2\right)J_{2}\left(m_{0}\right)$ is established by adjusting $m_{0}$ and $\varphi_{0}$, five optical comb teeth with equal output amplitude can be obtained, as shown in the following equation:

(2) $$E_{5-OFC}\left(t\right)=\sum_{n=1}^{5}A_{n}e^{jn\omega_{0}t}$$

where $A_{n}\left(n=1\ldots 5\right)$ represents the magnitude of each comb tooth, and $\omega_{0}$ is the comb spacing.

Next, the five-line OFC is divided by the interleaver into three-line OFCs and two-line OFCs with equal frequency intervals, whose expressions are as follows:

(3) $$E_{3-OFC}\left(t\right)=\sum_{n=1,3,5}A_{n}e^{jn\omega_{0}t}=A_{1}e^{j\omega_{0}t}+A_{3}e^{j3\omega_{0}t}+A_{5}e^{j5\omega_{0}t}$$

(4) $$E_{2-OFC}\left(t\right)=\sum_{n=2,4}A_{n}e^{jn\omega_{0}t}=A_{2}e^{j2\omega_{0}t}+A_{4}e^{j4\omega_{0}t}$$

Assuming that the desired signal, the direct path SI signal of this antenna, and the cross-path interference signal of the adjacent antennas received by antenna 1 and antenna 2 are as follows:

(5) $$\begin{array}{c} V_{RF1}\left(t\right)=V_{RF1}\cos\left[\omega_{RF1}\left(t+\tau_{1}\right)\right]=V_{RF1}\cos\left(\omega_{RF1}t+\theta_{1}\right)\\ V_{RF2}\left(t\right)=V_{RF2}\cos\left[\omega_{RF2}\left(t+\tau_{2}\right)\right]=V_{RF2}\cos\left(\omega_{RF2}t+\theta_{2}\right)\\ V_{SI11}\left(t\right)=V_{11}\cos\left[\omega_{RF1}\left(t+\tau_{11}\right)\right]=V_{11}\cos\left(\omega_{RF1}t+\theta_{11}\right)\\ V_{SI22}\left(t\right)=V_{22}\cos\left[\omega_{RF2}\left(t+\tau_{22}\right)\right]=V_{22}\cos\left(\omega_{RF2}t+\theta_{22}\right)\\ V_{SI12}\left(t\right)=V_{12}\cos\left[\omega_{RF1}\left(t+\tau_{12}\right)\right]=V_{12}\cos\left(\omega_{RF1}t+\theta_{12}\right)\\ V_{SI21}\left(t\right)=V_{21}\cos\left[\omega_{RF2}\left(t+\tau_{21}\right)\right]=V_{21}\cos\left(\omega_{RF2}t+\theta_{21}\right) \end{array}$$

where $\omega_{RF1}$ and $\omega_{RF2}$ represent the angular frequencies of the RF1 and RF2 signals, respectively. $V_{RF1}$, $V_{RF2}$, $V_{11}$, $V_{22}$, $V_{12}$, and $V_{21}$ are the amplitudes of the desired signals, RF1 and RF2, and interference signals SI11, SI22, SI12, and SI21, respectively. $\tau_{1}$ and $\tau_{2}$ represent the delay experienced by the desired signals RF1 and RF2 from transmission to reception. $\tau_{11}$, $\tau_{22}$, $\tau_{12}$, and $\tau_{21}$ represent the delays experienced by the interference signal from transmitting antenna 1 to receiving antenna 1, transmitting antenna 2 to receiving antenna 2, transmitting antenna 1 to receiving antenna 2, and transmitting antenna 2 to receiving antenna 1, respectively. $\theta_{1}$, $\theta_{2}$, $\theta_{11}$, $\theta_{22}$, $\theta_{12}$, and $\theta_{21}$ indicate the equivalent phase shift of the delay of the above signal in the single-frequency or narrowband case.

In this scheme, the PDM-MZM implements the dual-polarization DSB modulation function. The mixed signal $V_{RF1}\left(t\right)+V_{SI11}\left(t\right)+V_{SI21}\left(t\right)$ drives the upper sub-modulator of the PDM-MZM, and the mixed signal $V_{RF2}\left(t\right)+V_{SI22}\left(t\right)+V_{SI12}\left(t\right)$ drives the lower sub-modulator of the PDM-MZM, both of which operate in quadrature points. The polarization multiplexed optical signal output by PDM-MZM can be expressed as follows:

(6) $$E_{PDM-MZM}\left(t\right)\approx \frac{\sqrt{2\mu_{1}}}{2}\left[\begin{array}{c} \sum_{n=1}^{5}A_{n}e^{jn\omega_{0}t}\left\{\begin{array}{l} J_{0}\left(m_{1}\right)J_{0}\left(m_{11}\right)J_{0}\left(m_{21}\right)\\ -\left[\begin{array}{l} J_{0}\left(m_{1}\right)J_{0}\left(m_{11}\right)J_{1}\left(m_{21}\right)\left(\begin{array}{l} e^{j\omega_{RF2}t+j\theta_{21}}\\ +e^{-j\omega_{RF2}t-j\theta_{21}} \end{array}\right)\\ +J_{0}\left(m_{1}\right)J_{1}\left(m_{11}\right)J_{0}\left(m_{21}\right)\left(\begin{array}{l} e^{j\omega_{RF1}t+j\theta_{11}}\\ +e^{-j\omega_{RF1}t-j\theta_{11}} \end{array}\right)\\ +J_{1}\left(m_{1}\right)J_{0}\left(m_{11}\right)J_{0}\left(m_{21}\right)\left(\begin{array}{l} e^{j\omega_{RF1}t+j\theta_{1}}\\ +e^{-j\omega_{RF1}t-j\theta_{1}} \end{array}\right) \end{array}\right] \end{array}\right\}\cdot {\vec{e}}_{TE}\\ \sum_{n=1}^{5}A_{n}e^{jn\omega_{0}t}\left\{\begin{array}{l} J_{0}\left(m_{2}\right)J_{0}\left(m_{22}\right)J_{0}\left(m_{12}\right)\\ -\left[\begin{array}{l} J_{0}\left(m_{2}\right)J_{0}\left(m_{22}\right)J_{1}\left(m_{12}\right)\left(\begin{array}{l} e^{j\omega_{RF1}t+j\theta_{12}}\\ +e^{-j\omega_{RF1}t-j\theta_{12}} \end{array}\right)\\ +J_{0}\left(m_{2}\right)J_{1}\left(m_{22}\right)J_{0}\left(m_{12}\right)\left(\begin{array}{l} e^{j\omega_{RF2}t+j\theta_{22}}\\ +e^{-j\omega_{RF2}t-j\theta_{22}} \end{array}\right)\\ +J_{1}\left(m_{2}\right)J_{0}\left(m_{22}\right)J_{0}\left(m_{12}\right)\left(\begin{array}{l} e^{j\omega_{RF2}t+j\theta_{2}}\\ +e^{-j\omega_{RF2}t-j\theta_{2}} \end{array}\right) \end{array}\right] \end{array}\right\}\cdot {\vec{e}}_{TM} \end{array}\right]$$

where $\mu_{1}$ is the insertion loss of PDM-MZM, and ${\vec{e}}_{TE}$ and ${\vec{e}}_{TM}$ represent the unit vectors of the TE- and TM mode of the light field. PC1 and PBS demultiplex the above signals, and the output signals of the two sub-modulators of the PDM-MZM are split into two paths and fed into BPD1 and BPD2, respectively. The photocurrents of the two mixed received signals are obtained as follows:

(7) $$i_{BPD11}\left(t\right)\approx \eta_{1}\mu_{1}\sum_{n=1}^{5}A_{n}^{2}\left\{\begin{array}{l} DC+HMD\\ -\frac{1}{2}\left[\begin{array}{l} J_{0}^{2}\left(m_{1}\right)J_{0}^{2}\left(m_{11}\right)J_{0}\left(m_{21}\right)J_{1}\left(m_{21}\right)\cos\left(\omega_{RF2}t+\theta_{21}\right)\\ +J_{0}^{2}\left(m_{1}\right)J_{0}\left(m_{11}\right)J_{1}\left(m_{11}\right)J_{0}^{2}\left(m_{21}\right)\cos\left(\omega_{RF1}t+\theta_{11}\right)\\ +J_{0}\left(m_{1}\right)J_{1}\left(m_{1}\right)J_{0}^{2}\left(m_{11}\right)J_{0}^{2}\left(m_{21}\right)\cos\left(\omega_{RF1}t+\theta_{1}\right) \end{array}\right] \end{array}\right\}$$

(8) $$i_{BPD21}\left(t\right)\approx \eta_{2}\mu_{1}\sum_{n=1}^{5}A_{n}^{2}\left\{\begin{array}{l} DC+HMD\\ -\frac{1}{2}\left[\begin{array}{l} J_{0}^{2}\left(m_{2}\right)J_{0}^{2}\left(m_{22}\right)J_{0}\left(m_{12}\right)J_{1}\left(m_{12}\right)\cos\left(\omega_{RF1}t+\theta_{12}\right)\\ +J_{0}^{2}\left(m_{2}\right)J_{0}\left(m_{22}\right)J_{1}\left(m_{22}\right)J_{0}^{2}\left(m_{12}\right)\cos\left(\omega_{RF2}t+\theta_{22}\right)\\ +J_{0}\left(m_{2}\right)J_{1}\left(m_{2}\right)J_{0}^{2}\left(m_{22}\right)J_{0}^{2}\left(m_{12}\right)\cos\left(\omega_{RF2}t+\theta_{2}\right) \end{array}\right] \end{array}\right\}$$

where $\eta_{1}$ and $\eta_{2}$ represent the responsiveness of the two BPDs. According to Equations (7) and (8), when no interference cancellation process is performed, the desired received signal is contaminated.

Then, the analog reconstruction of the reference signal is performed. First, a suitable SSB modulation method must be chosen to modulate the reference interference signal. Traditional SSB modulation methods can be divided into three types: those based on optical filters, those based on optical I/Q modulators, and those based on DDMZM [28]. A comprehensive analysis of the characteristics of the three methods will be given in the subsequent section of the Discussion. Here, SSB modulation based on optical I/Q modulator will be utilized as a case study for mathematical derivation in this context.

Assume that the two initial reference interference signals are respectively expressed as follows:

(9) $$\begin{array}{c} V_{R1}\left(t\right)=V_{R1}\cos\left(\omega_{RF1}t\right)\\ V_{R2}\left(t\right)=V_{R2}\cos\left(\omega_{RF2}t\right) \end{array}$$

$V_{R1}\left(t\right)$ drives I/Q modulator1 (essentially a quadrature point-biased DPMZM) after passing through a 90° bridge, and $V_{R2}\left(t\right)$ drives I/Q modulator2 after passing through a 90° bridge. The optical SSB output expression is expressed as follows:

(10) $$E_{SSBi}\left(t\right)\approx \frac{\sqrt{2}}{4}\sqrt{\mu_{i}}E_{2/3-OFC}\left(t\right)\left[\left(1-j\right)J_{0}\left(m_{ri}\right)-2J_{1}\left(m_{ri}\right)e^{j\omega_{RFi}t}\right]$$

where $\mu_{i}\left(i=2,3\right)$ represents the losses of the two I/Q modulators, and $m_{ri}\left(i=1,2\right)$ represents the modulation index of the two modulators.

Next, PC2 is adjusted so that the two SSB signals have orthogonal polarization states after PBC coupling and obtain a polarization multiplexed optical signal, as follows:

(11) $$E_{PBC}\left(t\right)=\frac{\sqrt{2}}{4}\left[\begin{array}{c} \sqrt{\mu_{2}}\sum_{n=1,3,5}\left\{A_{n}e^{jn\omega_{0}t}\left[\left(1-j\right)J_{0}\left(m_{r1}\right)-2J_{1}\left(m_{r1}\right)e^{j\omega_{RF1}t}\right]\right\}\cdot {\vec{e}}_{TE}\\ \sqrt{\mu_{3}}\sum_{n=2,4}\left\{A_{n}e^{jn\omega_{0}t}\left[\left(1-j\right)J_{0}\left(m_{r2}\right)-2J_{1}\left(m_{r2}\right)e^{j\omega_{RF2}t}\right]\right\}\cdot {\vec{e}}_{TM} \end{array}\right]$$

When the above signals are input to the programmable spectrum processor for attenuation, phase shifting, and filtering [29,30], the two reconstructed reference optical signals are as follows:

(12) $$E_{CH1}\left(t\right)=\frac{\sqrt{2}}{4}\left[\begin{array}{c} \sqrt{\mu_{2}}A_{1}e^{j\omega_{0}t}\left[\begin{array}{l} \left(1-j\right)J_{0}\left(m_{r1}\right)\\ -2\alpha_{1}J_{1}\left(m_{r1}\right)e^{j\left(\omega_{RF1}t+\beta_{1}\right)} \end{array}\right]\cdot {\vec{e}}_{TE}\\ \sqrt{\mu_{3}}A_{2}e^{j2\omega_{0}t}\left[\begin{array}{l} \left(1-j\right)J_{0}\left(m_{r2}\right)\\ -2\alpha_{2}J_{1}\left(m_{r2}\right)e^{j\left(\omega_{RF2}t+\beta_{2}\right)} \end{array}\right]\cdot {\vec{e}}_{TM} \end{array}\right]$$

(13) $$E_{CH2}\left(t\right)=\frac{\sqrt{2}}{4}\left[\begin{array}{c} \sqrt{\mu_{2}}A_{3}e^{j3\omega_{0}t}\left[\begin{array}{l} \left(1-j\right)J_{0}\left(m_{r1}\right)\\ -2\alpha_{3}J_{1}\left(m_{r1}\right)e^{j\left(\omega_{RF1}t+\beta_{3}\right)} \end{array}\right]\cdot {\vec{e}}_{TE}\\ \sqrt{\mu_{3}}A_{4}e^{j4\omega_{0}t}\left[\begin{array}{l} \left(1-j\right)J_{0}\left(m_{r2}\right)\\ -2\alpha_{4}J_{1}\left(m_{r2}\right)e^{j\left(\omega_{RF2}t+\beta_{4}\right)} \end{array}\right]\cdot {\vec{e}}_{TM} \end{array}\right]$$

where $\alpha_{i}\left(i=1,2,3,4\right)$ represents the attenuation coefficient, and $\beta_{i}\left(i=1,2,3,4\right)$ represents the phase shift. Based on Finisar’s high-resolution, solid-state Liquid Crystal on Silicon (LCoS) optical engine, the reconfigurable optical processor (WaveShaper 16000A, Finisar, San Jose, CA, USA) covers the entire C-band, providing precise control of the filter wavelength, bandwidth, shape, and phase, as well as the ability to switch and combine multiple signals. Inputting the two above optical signals into BPD1 and BPD2, the two reconstructed reference currents can be obtained as follows:

(14) $$i_{BPD12}\left(t\right)\approx \frac{\sqrt{2}}{2}\eta_{1}\left\{\begin{array}{l} \mu_{2}A_{1}^{2}\left[J_{0}^{2}\left(m_{r1}\right)+2\alpha_{1}^{2}J_{1}^{2}\left(m_{r1}\right)\right]\\ +\mu_{3}A_{2}^{2}\left[J_{0}^{2}\left(m_{r2}\right)+2\alpha_{2}^{2}J_{1}^{2}\left(m_{r2}\right)\right]\\ -\mu_{2}A_{1}^{2}\sqrt{2}\alpha_{1}J_{0}\left(m_{r1}\right)J_{1}\left(m_{r1}\right)\cos\left(\omega_{RF1}t-\pi /4+\beta_{1}\right)\\ -\mu_{3}A_{2}^{2}\sqrt{2}\alpha_{2}J_{0}\left(m_{r2}\right)J_{1}\left(m_{r2}\right)\cos\left(\omega_{RF2}t-\pi /4+\beta_{2}\right) \end{array}\right\}$$

(15) $$i_{BPD22}\left(t\right)\approx \frac{\sqrt{2}}{2}\eta_{2}\left\{\begin{array}{l} \mu_{2}A_{3}^{2}\left[J_{0}^{2}\left(m_{r1}\right)+2\alpha_{3}^{2}J_{1}^{2}\left(m_{r1}\right)\right]\\ +\mu_{3}A_{4}^{2}\left[J_{0}^{2}\left(m_{r2}\right)+2\alpha_{4}^{2}J_{1}^{2}\left(m_{r2}\right)\right]\\ -\mu_{2}A_{3}^{2}\sqrt{2}\alpha_{3}J_{0}\left(m_{r1}\right)J_{1}\left(m_{r1}\right)\cos\left(\omega_{RF1}t-\pi /4+\beta_{3}\right)\\ -\mu_{3}A_{4}^{2}\sqrt{2}\alpha_{4}J_{0}\left(m_{r2}\right)J_{1}\left(m_{r2}\right)\cos\left(\omega_{RF2}t-\pi /4+\beta_{4}\right) \end{array}\right\}$$

When the programmable spectral processor is controlled to adjust the attenuation and phase shift to satisfy the amplitude and phase matching conditions shown in the following equation, the final pure signals output from BPD1 and BPD2 can be expressed as shown in Equations (17) and (18).

(16) $$\left\{\begin{array}{l} \eta_{1}\mu_{1}\sum_{n=1}^{5}\left[A_{n}^{2}J_{0}^{2}\left(m_{1}\right)J_{0}\left(m_{11}\right)J_{1}\left(m_{11}\right)J_{0}^{2}\left(m_{21}\right)\right]=\sqrt{2}\eta_{1}\mu_{2}A_{1}^{2}\alpha_{1}J_{0}\left(m_{r1}\right)J_{1}\left(m_{r1}\right)\\ \eta_{1}\mu_{1}\sum_{n=1}^{5}\left[A_{n}^{2}J_{0}^{2}\left(m_{1}\right)J_{0}^{2}\left(m_{11}\right)J_{0}\left(m_{21}\right)J_{1}\left(m_{21}\right)\right]=\sqrt{2}\eta_{1}\mu_{3}A_{2}^{2}\alpha_{2}J_{0}\left(m_{r2}\right)J_{1}\left(m_{r2}\right)\\ \eta_{2}\mu_{1}\sum_{n=1}^{5}\left[A_{n}^{2}J_{0}^{2}\left(m_{2}\right)J_{0}\left(m_{22}\right)J_{1}\left(m_{22}\right)J_{0}^{2}\left(m_{12}\right)\right]=\sqrt{2}\eta_{2}\mu_{2}A_{4}^{2}\alpha_{4}J_{0}\left(m_{r2}\right)J_{1}\left(m_{r2}\right)\\ \eta_{2}\mu_{1}\sum_{n=1}^{5}\left[A_{n}^{2}J_{0}^{2}\left(m_{2}\right)J_{0}^{2}\left(m_{22}\right)J_{0}\left(m_{12}\right)J_{1}\left(m_{12}\right)\right]=\sqrt{2}\eta_{2}\mu_{3}A_{3}^{2}\alpha_{3}J_{0}\left(m_{r1}\right)J_{1}\left(m_{r1}\right)\\ \theta_{11}=-\pi /4+\beta_{1}\pm 2k\pi \\ \theta_{21}=-\pi /4+\beta_{2}\pm 2k\pi \\ \theta_{22}=-\pi /4+\beta_{4}\pm 2k\pi \\ \theta_{12}=-\pi /4+\beta_{3}\pm 2k\pi \end{array}\right.$$

(17) $$i_{BPD1}\left(t\right)\approx \eta_{1}\mu_{1}\sum_{n=1}^{5}A_{n}^{2}\left[-2J_{0}\left(m_{1}\right)J_{1}\left(m_{1}\right)J_{0}^{2}\left(m_{11}\right)J_{0}^{2}\left(m_{21}\right)\cos\left(\omega_{RF1}t+\theta_{1}\right)\right]$$

(18) $$i_{BPD2}\left(t\right)\approx \eta_{2}\mu_{1}\sum_{n=1}^{5}A_{n}^{2}\left[-2J_{0}\left(m_{2}\right)J_{1}\left(m_{2}\right)J_{0}^{2}\left(m_{22}\right)J_{0}^{2}\left(m_{12}\right)\cos\left(\omega_{RF2}t+\theta_{2}\right)\right]$$

In summary, when the programmable spectrum processor is adjusted to satisfy amplitude and phase matching conditions, and the phase reversal is implemented in the BPD, both SI signals received by each antenna can be canceled, and only the desired received signal is retained.

## 3. Simulation and Results

The VPI simulation test platform was built according to Figure 1, and the device parameter settings in the simulation were aligned with the actual device parameters (KG-TSL-C-13, Conquer, Beijing, China; FTM7938EZ, Fujitsu, Kawasaki, Japan; FTM7961 Fujitsu, Kawasaki, Japan; FTM7981 Fujitsu, Kawasaki, Japan; UFP Optical Interleaver GouMax, Milpitas, CA, USA; CPB30 Keopsys, Lannion, France; DSC720, Discovery Semiconductor, Ewing Township, NJ, USA; Waveshaper16000A, Finisar, San Jose, CA, USA). A laser source with a power of 16 dBm, a center wavelength of 1552.5 nm, and an RIN of −145 dBc/Hz is set as the optical carrier. The optical signal input into the MZM is modulated by an RF signal with a frequency of 40 GHz. It is used as an OFC generator module to generate an OFC signal. The half-wave voltage of the MZM is 3.5 V, the insertion loss is 8 dB, and the extinction ratio is about 25 dB. Then, the OFC signal passes through the 50/50 optical beam splitter and is sent to the PDM-MZM. The half-wave voltage is 3.5 V, the insertion loss is 10 dB, and the extinction ratio is about 25 dB. Meanwhile, the lower channel is sent to the interleaver, and the channel bandwidth is 25 GHz. The two output optical signals of the interleaver are input into DPMZM1 and DPMZM2, and are modulated by the reference signal. The half-wave voltage of the DPMZM is 3.5 V, the insertion loss is 6 dB, and the extinction ratio is about 25 dB. Subsequently, an erbium-doped optical fiber amplifier (EDFA) with a noise figure (NF) less than 5 dB is added after the PC1 and PBC for optical power compensation. The programmable spectrum processor works in 1 × N mode and simultaneously implements filtering, attenuation, and phase-shifting operations. Finally, the upper channel receives an optical signal, and the lower channel reference optical signal is sent to the BPD with a responsivity of 0.65 A/W for photoelectric detection, and is observed with a signal analyzer.

**Figure 2:**
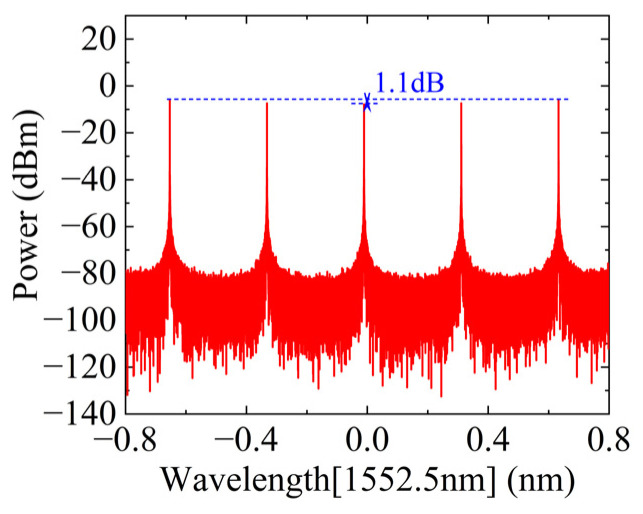
The spectra of the five-line OFC.

**Figure 3:**
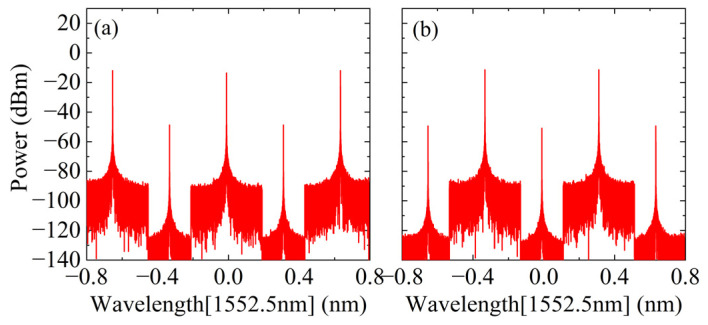
The spectra of the OFCs: (**a**) three-line; (**b**) two-line.

First, the modulation index, $m_{0}=1.703$, and DC bias angle, $\varphi_{0}$, of the MZM are adjusted to output a five-line OFC signal, as shown in Figure 2. The maximum amplitude fluctuation between the five comb teeth is about 1.1 dB, and the overall amplitude is relatively flat. As precise optical amplitude attenuation adjustment is required in the subsequent programmable spectrum processor, the amplitude fluctuation here will not affect the final SIC effect. Then, the above five-line OFC is input into the interleaver for channel division, and the three-line OFC and two-line OFC are obtained as shown in Figure 3a,b, respectively. To conform to the parameters of the real device, the optical filter type is configured as rectangular in the simulation, resulting in a channel isolation of approximately 35 dB. Notably, according to the working process of the programmable spectrum processor in Figure 1, the initial five-line OFC can be replaced by a four-line OFC without affecting the system function. Therefore, the three-line OFC shown in Figure 3a can also be replaced by a two-line OFC.

Next, to distinguish the desired signals of the two antennas, the desired signal frequencies of antennas 1 and 2 are set to 24 GHz and 24.5 GHz, respectively. The SI signal received by this antenna with a frequency of 24 GHz and a power of −10 dBm and the SI signal received by an adjacent antenna with a frequency of 24.5 GHz and a power of −13 dBm are coupled and received by antenna 1 and drive the upper sub-modulator of PDM-MZM. The SI signal received by this antenna with a frequency of 24.5 GHz and a power of −10 dBm and the SI signal received by an adjacent antenna with a frequency of 24 GHz and a power of −13 dBm are coupled and received by antenna 2, to drive the lower sub-modulator of the PDM-MZM. Both sub-modulators of the PDM-MZM work at quadrature points. Then, the reference interference signal with a frequency of 24 GHz and a power of 10 dBm drives DPMZM1, and the reference interference signal with a frequency of 24.5 GHz and a power of 10 dBm drives DPMZM2. The optical signals of the reference path are all SSB-modulated. After that, the programmable spectrum processor performs filtering, attenuation, and phase-shifting operations at specific frequencies, and the amplitude and phase matching of the reference interference signal is achieved. Finally, the SIC can be completed after the inversion processing of the BPD. It should be noted that there is no packaged programmable spectrum processing module in VPI; we need to build it ourselves according to the principle. In order to simplify the simulation structure, the optical filter group, the tunable optical attenuator group, and the optical phase shifter group are used instead. Additionally, from a theoretical perspective, whether the desired received signal is applied does not affect the SIC process. Therefore, the desired received signal is not applied here to simplify the simulation test. It is imperative to emphasize that the desired received signal must exist in practical scenarios, which will introduce additional DC or spurious components after beating with the residual interference signal. However, these components can be filtered out by photodetectors with direct-blocking devices or filters.

**Figure 4:**
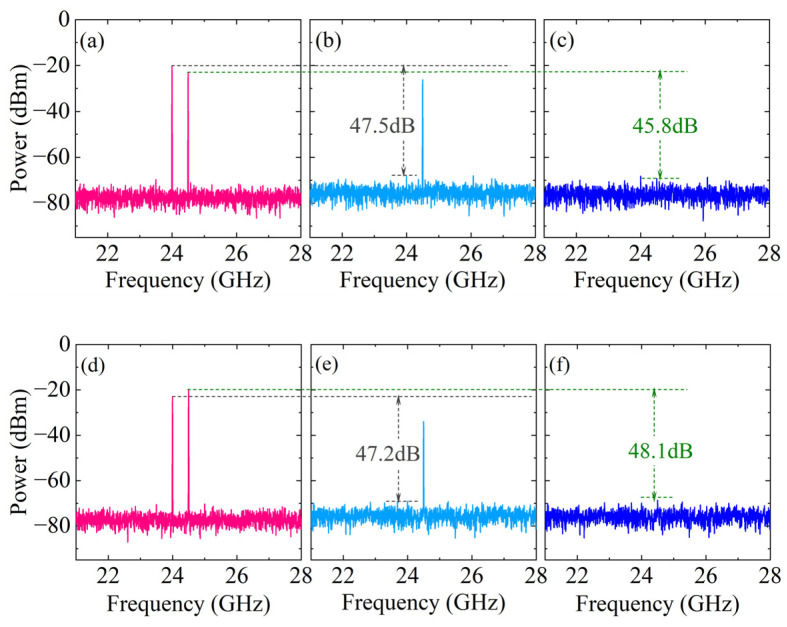
The output single-tone spectra (**a**–**c**) without/with SIC at antenna 1; (**d**–**f**) without/with SIC at antenna 2; (**a**,**d**) without SIC; (**b**,**e**) with single-path SIC; and (**c**,**f**) with dual-path SIC.

The single-frequency spectrum before and after SIC is shown in Figure 4a–f. Among them, the output spectrum before SIC at antenna 1 is shown in Figure 4a, and obvious SI signals introduced by the two antennas can be observed. Under the predetermined signal frequency and power settings, when $\tau_{11}=\tau_{22}=\tau_{21}=\tau_{12}=10ps$, the amplitude attenuation coefficients $\alpha_{1}\approx 0.415$, $\alpha_{2}\approx 0.344$, $\alpha_{3}\approx 0.427$, $\alpha_{4}\approx 0.688$ and phase shift $\beta_{1}\approx 44.7^{o}$, $\beta_{2}\approx -42.1^{o}$, $\beta_{3}\approx 45.4^{o}$, $\beta_{4}\approx -46.8^{o}$ are determined. In addition, according to the WaveShaper 16000A data manual, the bandwidth of the optical filter is set to 10 GHz. As shown in Figure 4b, after single-path SIC processing, the SI signal introduced by this antenna is canceled, and the SIC depth is approximately 47.5 dB. However, there are still residual SI signals from the adjacent antenna. Then, the programmable spectrum processor is controlled to perform further SIC processing, and the output spectrum is obtained, as shown in Figure 4c, with an SIC depth of approximately 45.8 dB. The output spectra before and after the SIC at antenna 2 are shown in Figure 4d–f; the SIC depths under the two paths are 48.1 dB and 47.2 dB, respectively. Due to the limitation of path loss, the SIC depth of the adjacent antenna SI path is slightly lower.

**Figure 5:**
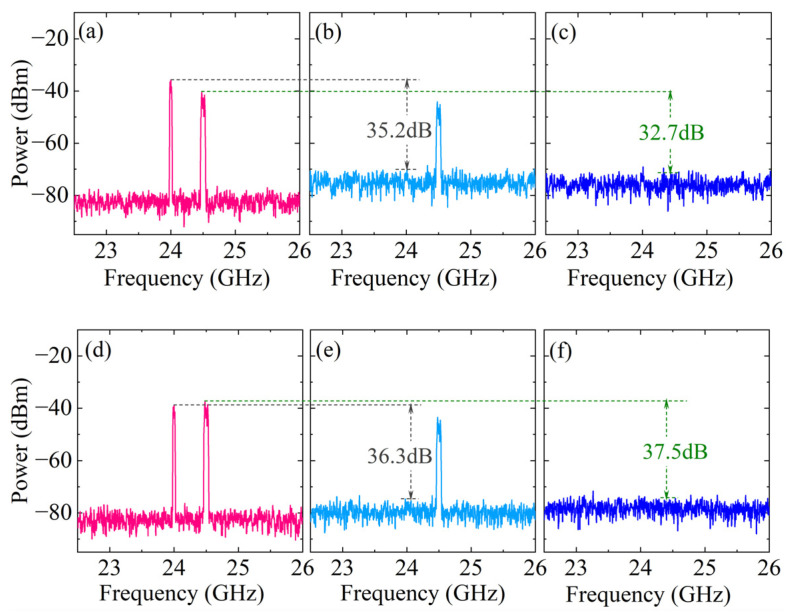
The output 30/60 MHz narrowband spectra (**a**–**c**) without/with SIC at antenna 1; (**d**–**f**) without/with SIC at antenna 2; (**a**,**d**) without SIC; (**b**,**e**) with single-path SIC; and (**c**,**f**) with dual-path SIC.

Then, the narrowband SIC characteristics of the system are verified. The SI signal received by antenna 1 is changed to a 16QAM signal with a carrier frequency of 24 GHz, a power of −20 dBm, and a bandwidth of 30 MHz. The SI signal received by antenna 2 is changed to a 16QAM signal with a carrier frequency of 24.5 GHz, a power of −23 dBm, and a bandwidth of 60 MHz. The power of the reference interference signal is −10 dBm, and the other parameters are the same as the received interference signal. Based on the previous parameters, the amplitude attenuation coefficient and phase shift are adjusted appropriately; Figure 5a–c show the output spectra before and after SIC at antenna 1. After dual-path SIC processing, the narrowband SIC depths are 35.2 dB and 32.7 dB. Figure 5d–f show the output spectrum before and after SIC at antenna 2. The narrowband SIC depths are 37.5 dB and 36.3 dB, respectively. Since the reconstruction path of the reference interference signal is more complex than the receiving path and introduces more attenuation, the noise floor is slightly higher after optical power compensation by EDFA.

**Figure 6:**
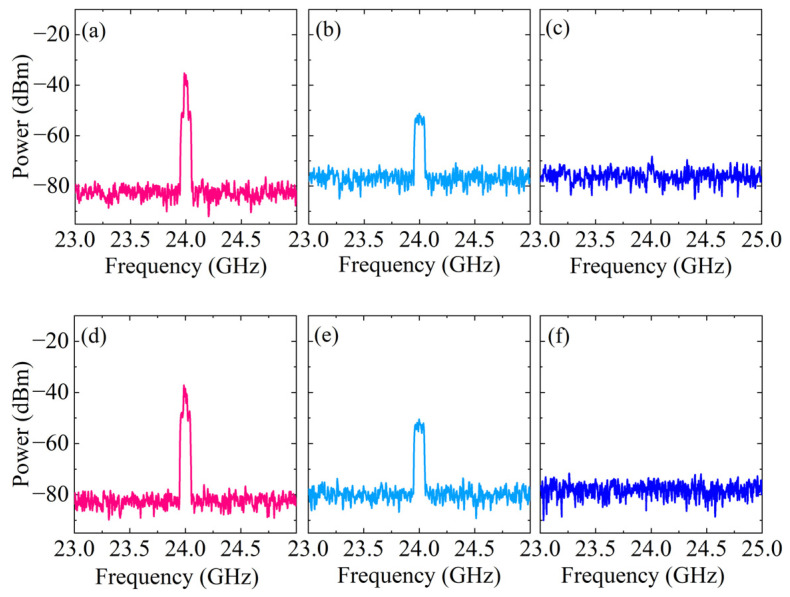
The output 30/90 MHz narrowband spectra (**a**–**c**) without/with SIC at antenna 1; (**d**–**f**) without/with SIC at antenna 2; (**a**,**d**) without SIC; (**b**,**e**) with single-path SIC; and (**c**,**f**) with dual-path SIC.

In certain IBFD–MIMO application scenarios, multiple antennas’ desired signal carrier frequencies are similar or equal, but the bandwidths or modulation formats are different. Based on this scenario, the received SI signal of antenna 1 is kept unchanged, and the received SI signal of antenna 2 is changed to a 64QAM signal with a carrier frequency of 24 GHz, a power of −23 dBm, and a bandwidth of 90 MHz. The power of the reference interference signal is still −10 dBm, and other parameters change synchronously according to the received interference signal. The output spectra before and after SIC at antenna 1 and antenna 2 are shown in Figure 6a–c and Figure 6d–f, respectively. It can be found that the SIC processing of this scheme has nothing to do with the format of the interference signal. After dual-path SIC processing, the SI signals of the two antennas are canceled.

**Figure 7:**
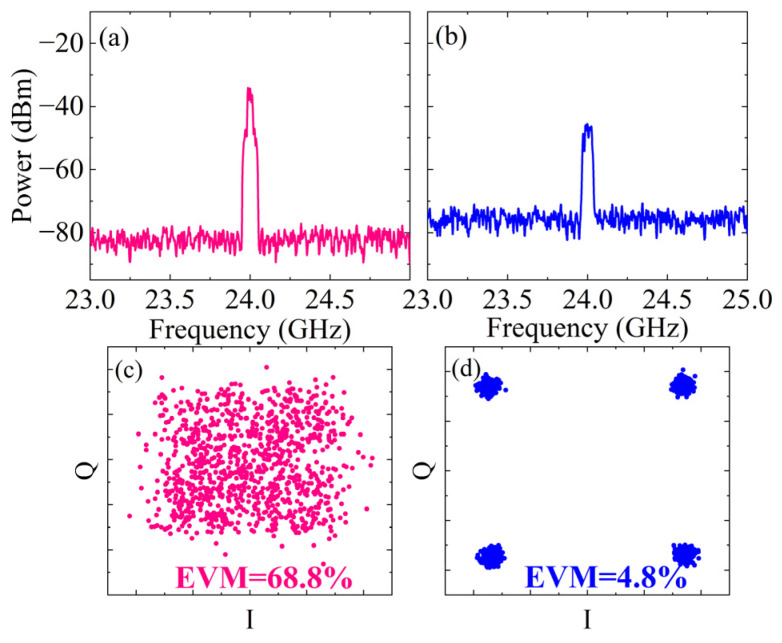
Antenna 1, after adding the desired signal (4QAM, 70 MHz). (**a**,**b**) Output narrowband spectrum without/with SIC; (**c**,**d**) demodulation constellation diagram without/with SIC.

Finally, in order to verify the receiving performance of this solution, the desired received signal is added based on the parameter settings in Figure 6. Among them, the carrier frequency is 24 GHz, the power is −30 dBm, the bandwidth is 70 MHz, and the modulation format is 4QAM. The desired signal is mixed with the received SI signal and then modulated. The output spectrum before and after SIC at antenna 1 is shown in Figure 7a,b. After dual-path SIC processing, only the desired received signal remains in the spectrum. The constellation diagram before SIC is shown in Figure 7c. When signals of multiple modulation formats are mixed, the demodulator may erroneously demodulate some symbols into symbols of another modulation method, leading to a misjudgment of constellation points. Consequently, the constellation diagram appears disorganized, with an error vector magnitude (EVM) of 68.8%, and the communication system cannot work correctly due to complete bit errors. The spectrum after SIC is shown in Figure 7b, where both the interference signal introduced by antenna 1 itself and introduced by antenna 2 to antenna 1 are effectively eliminated, leaving only the desired 4QAM signal. The demodulator has achieved correct demodulation, making the four constellation points visible. Figure 7d shows that the EVM is only 4.8%, far below the communication standard requirements. This indicates that the proposed SIC method enhances the demodulation ability of the desired signal at the receiving terminal, enabling it to identify and demodulate the desired signal. Consequently, this reduces error rates, thereby enhancing communication quality and reliability more effectively.

**Figure 8:**
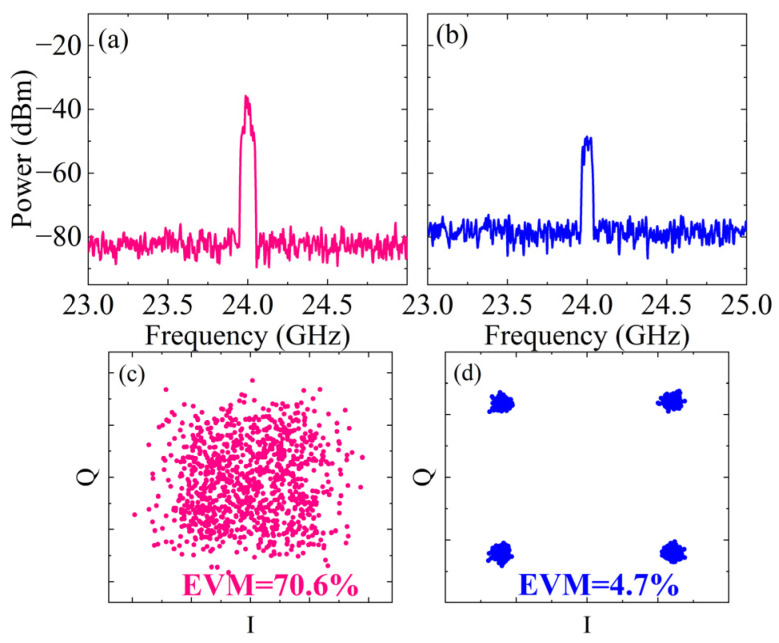
Antenna 2, after adding the desired signal (4QAM, 70 MHz). (**a**,**b**) Output narrowband spectrum without/with SIC; (**c**,**d**) demodulation constellation diagram without/with SIC.

Similarly, the output spectrum and constellation diagram before and after SIC at antenna 2 are shown in Figure 8a–d. After dual-path SIC processing, the EVM dropped from 70.6% to 4.7%, and system performance improved.

In summary, a comparison between the proposed scheme and some existing methods [16,17,20,21,26] was conducted regarding the single-frequency SIC depth, as shown in Table 1. Due to the absence of fiber and the reference branch being SSB-modulated, the SIC depth will not decrease at a specific frequency point, as mentioned in [16]. Moreover, compared with [17] and [21], the overall loss of the proposed scheme is larger after cascading the five-line OFC. Therefore, under the action of the optical amplifier with fixed power output, the noise floor is raised, resulting in the residual interference signal being submerged in the noise floor, and the single-frequency SIC depth is limited. Compared with the 3 × 3 MIMO system illustrated in [26], although the single-frequency SIC depth of the proposed scheme is slightly improved, the simulation verification consistently cannot be compared with the experimental test. According to [26], the reason for limiting SIC depth is the signal-to-noise ratio and amplitude/delay matching accuracy. Therefore, if there is an opportunity to conduct experimental testing on the proposed method in the future, our focus should be on the following:

(1) How to solve the significant loss issue in the system, improve the signal-to-noise ratio, and thereby enhance the SIC depth. In addition to optimizing the quality of the OFC, integration technology can be used in the future to further reduce the size and energy consumption of the system.
(2) How to optimize the amplitude/phase matching accuracy. Of course, this is related to the adjustment accuracy of the programmable spectrum processor. To reduce reliance on instrument performance, future considerations may include integrating SIC algorithms after photodetection to further enhance SIC performance.

**Table 1:** Comparison of the proposed scheme and some existing schemes.

Scheme	Single-Frequency SIC Depth (dB)	Experiment/Simulation	Single Channel/MIMO
Ref [16]	>26.5	Experiment	Single channel
Ref [17]	>50.9	Experiment	Single channel
Ref [20]	>39	Experiment	Single channel
Ref [21]	>60	Experiment	Single channel
Ref [26]	44.6	Experiment	MIMO
Proposed Scheme	>45.8	Simulation	MIMO

## 4. Discussion

### 4.1. Selection of the SSB Modulation Method

**Figure 9:**
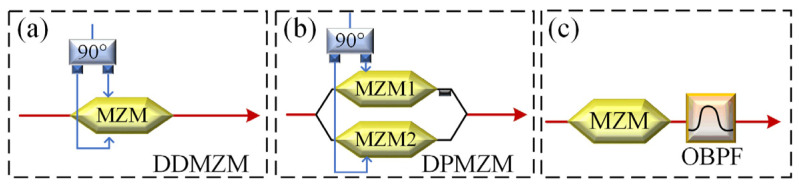
Schematic diagram of three SSB modulation structures based on lithium niobate modulators (**a**) based on DDMZM; (**b**) based on DPMZM; and (**c**) based on optical filter.

Electro-optic modulation is one of the essential components of microwave photonic systems, and its characteristics largely determine the performance of microwave photonic signal processing modules. In this study, the electro-optical modulation process of the reference signal was implemented based on the SSB modulation method. According to [28], conventional SSB modulation methods based on lithium niobate modulators can be divided into three types: those based on DDMZM, those based on DPMZM, and those based on optical filtering. The specific implementation principle is shown in Figure 9.

**Figure 10:**
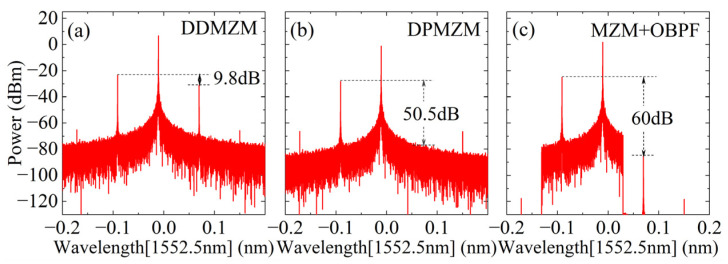
Three SSB modulation spectra (**a**) based on DDMZM; (**b**) based on DPMZM; and (**c**) based on optical filter.

Next, the characteristics of the three above SSB modulation methods are analyzed based on a simple simulation demonstration. The parameters of lasers, DDMZM, DPMZM, MZM, and OBPF used in the simulation are all aligned with the actual devices (KG-TSL-C-13, Conquer, Beijing, China; FTM7937EZ, Fujitsu, Kawasaki, Japan; FTM7961, FTM7938EZ, Fujitsu, Kawasaki, Japan; XTM-50, EXFO, Quebec City, QC, Canada). The frequency of the RF signal is set to 10 GHz, and the power is −10 dBm. The output spectra for the three SSB modulation modes are shown in Figure 10a–c. From the sideband suppression ratio (SSR) perspective, the SSB method based on DDMZM is the worst, with an SSR of only 9.8 dB, while the SSRs of the other two methods are 50.5 dB and 60 dB, respectively. In comparison, the spectra under the first two methods will have undesired sidebands remaining, while those under the third method are purer. However, the third method relies on the performance of the optical filter, which requires a higher out-of-band suppression ratio and a sharper roll-off. From the perspective of system bandwidth, thanks to the large operating bandwidth characteristics of RF devices and electro-optical modulators, the first two methods can easily tune the operating frequency. Meanwhile, the third method requires manual adjustment of the filter bandwidth, and the filter bandwidth often limits the operating frequency range of the system. From the perspective of implementation complexity, the first two methods both require additional 90° bridges and strict DC control, while the third method is relatively simple. However, due to the wide range of engineering application requirements, the SSB method based on DPMZM has gradually been commercialized and engineered. When used with the automatic bias control board [31], the system complexity is significantly reduced, and the system stability is enhanced. In summary, the SSB method based on DPMZM was used in this study.

### 4.2. Channel Expansion and Performance Optimization

**Figure 11:**
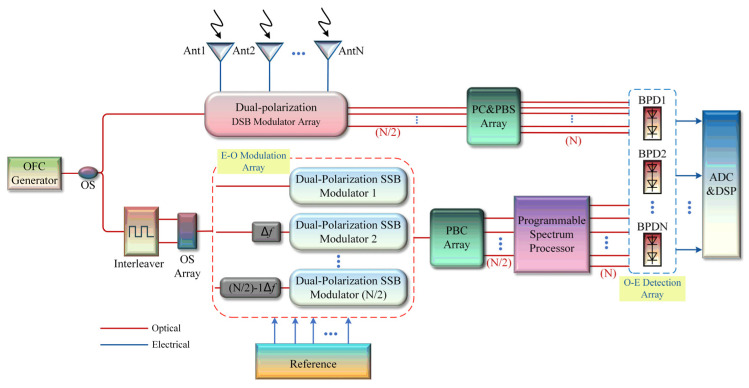
Schematic diagram of the improved microwave photonic SIC scheme for the N × N IBFD–MIMO communication system.

Massive MIMO technology has been widely studied and aims to mitigate the interference and fading effects during transmission, thereby improving a system’s adaptability to varying channel conditions. The proposed scheme has only been theoretically derived and simulated for 2 × 2 MIMO application scenarios. Although the functionality has been preliminarily confirmed, the scheme faces challenges amidst the evolving trend of antenna array expansion in the future. Therefore, for the N × N MIMO scenario, a possible microwave photonic SIC method is shown in Figure 11. Firstly, received N-channel signals are modulated in N/2 dual-polarization DSB modulators, and subsequently demultiplexed through PC and PBS arrays to obtain received N-channel optical signals. In the lower reference branch, (N/2-1) optical frequency shifter modules (frequency shift amount of Δf) are used to frequency-shift N/2 three-line OFC and two-line OFC and then modulate the reference interfering signals in N/2 dual-polarized SSB modulation modules. N/2 dual-polarization reference interference signals with different optical frequencies are inputted to the programmable spectrum processor to perform N-channel optical amplitude attenuation and phase shift adjustment. Finally, after N BPDs, pure N-channel receiving signals can be obtained. The structure shown in Figure 11 builds upon the framework presented in Figure 1, extending the pivotal modules into an array configuration. Thanks to the utilization of polarization multiplexing technology, the overall complexity of this scheme has been halved.

Nevertheless, numerous optoelectronic devices make the equipment more redundant as paths increase. Simultaneously, the computational complexity of the adaptive parameter control algorithm for amplitude and delay experiences exponential growth. To address the above challenges, the digital pre-distortion-based optical SIC scheme combines flexible digital processing with the broadband and high-frequency cancellation characteristics of microwave photonics, greatly simplifying the system structure. However, the digital pre-distortion method is limited by the receiving signal-to-noise ratio, adding analog–digital conversion/digital–analog conversion (ADC/DAC) dynamic range, etc. Although good SIC can be achieved in the optical domain, it will destroy the composition of the residual SI signal to a certain extent, making later digital domain processing difficult.

Furthermore, this paper emphasizes the analog domain SIC in MIMO systems. By eliminating strong SI signals superimposed on the desired received signal, the bit error rate can be reduced, and the quality of the received signal can be improved. With the same research objective, another method is to add a digital signal processing module to the receiving terminal, assisted by a performance optimization algorithm, which can also improve the receiving performance. In MIMO communication systems, conventional performance optimization algorithms include the maximum ratio combining algorithm [32], the uniform combining algorithm [33], and the minimum mean square error combining algorithm [34]. This type of algorithm makes full use of the channel gain differences between different antennas. The signal-to-noise ratio and system robustness can be effectively improved by weighing or selecting appropriate channel gains. It should be noted that these algorithms exhibit slight variations in weight allocation, channel estimation requirements, and sensitivity to system complexity. Choosing an appropriate algorithm usually requires the comprehensive consideration of specific communication scenarios and performance requirements. Therefore, by adding ADC and digital signal processing (DSP) modules to the system shown in Figure 11 and assisting the above algorithm, the performance of the N × N MIMO communication system based on microwave photonics can be multi-dimensionally optimized, which has significant reference value for improving the performance of large-scale MIMO communication systems.

## 5. Conclusions

In this paper, based on the application background of narrowband IBFD–MIMO communication, a method for implementing SIC in a narrowband 2 × 2 IBFD–MIMO communication system using microwave photonic technology is proposed. The five-line OFC is divided into a three-line OFC and two-line OFC through the interleaver, and a dual-polarization OFC with frequency shifting effect is constructed to achieve independent modulation of the dual-channel reference signal. To avoid spurious results, the reference signals are all SSB-modulated. Furthermore, the filtering, attenuation, and phase-shifting operations of the programmable spectral processor enable amplitude and phase matching, which, in turn, eliminates single-frequency and narrowband SI signals. The simulation results show that the single-frequency SIC depth at the 24 GHz frequency point exceeds 45.8 dB, and the narrowband SIC depth at the 30 MHz bandwidth exceeds 32.7 dB. Thanks to SIC, the desired signal with a modulation format of 4QAM can be demodulated to an EVM as low as 4.7%. The proposed scheme uses a single device to perform dual-channel amplitude and phase matching at the same time, and the complexity of the system is reduced. This is also the first exploration of the simultaneous elimination of dual SI sources in microwave photonics. After further channel expansion and performance optimization through auxiliary integrated devices and digital algorithms, this approach will have significant application value in the IoT, indoor navigation, positioning, and other fields.

## Data Availability

The raw data supporting the conclusions of this article will be made available by the authors on request.
